# Slow Recovery from Local Disturbances as an Indicator for Loss of Ecosystem Resilience

**DOI:** 10.1007/s10021-017-0154-8

**Published:** 2017-06-02

**Authors:** Ingrid A. van de Leemput, Vasilis Dakos, Marten Scheffer, Egbert H. van Nes

**Affiliations:** 1 Department of Environmental Sciences, Aquatic Ecology and Water Quality Management Group, Wageningen University, PO Box 47, 6700AA Wageningen, The Netherlands; 2 Institute of Integrative Biology, Adaptation to a Changing Environment, ETH Zurich, Zurich, Switzerland; 3 Institut des Sciences de l’Evolution de Montpellier (ISEM), BioDICe team, CNRS, Universite de Montpellier, Montpellier, France

**Keywords:** resilience, critical transition, recovery rate, alternative states, catastrophic shift, pulse experiment, landscape ecology

## Abstract

A range of indicators have been proposed for identifying the elevated risk of critical transitions in ecosystems. Most indicators are based on the idea that critical slowing down can be inferred from changes in statistical properties of natural fluctuations and spatial patterns. However, identifying these signals in nature has remained challenging. An alternative approach is to infer changes in resilience from differences in standardized experimental perturbations. However, system-wide experimental perturbations are rarely feasible. Here we evaluate the potential to infer the risk of large-scale systemic transitions from local experimental or natural perturbations. We use models of spatially explicit landscapes to illustrate how recovery rates upon small-scale perturbations decrease as an ecosystem approaches a tipping point for a large-scale collapse. We show that the recovery trajectory depends on: (1) the resilience of the ecosystem at large scale, (2) the dispersal rate of organisms, and (3) the scale of the perturbation. In addition, we show that recovery of natural disturbances in a heterogeneous environment can potentially function as an indicator of resilience of a large-scale ecosystem. Our analyses reveal fundamental differences between large-scale weak and local-scale strong perturbations, leading to an overview of opportunities and limitations of the use of local disturbance-recovery experiments.

**Electronic supplementary material:**

The online version of this article (doi:10.1007/s10021-017-0154-8) contains supplementary material, which is available to authorized users.

## Introduction

The idea that we might detect loss of resilience as an early-warning signal for critical transitions in ecological systems has attracted much attention (Scheffer and others 2012; Dakos and others 2015). It can be mathematically shown that close to a broad class of tipping points (namely zero-eigenvalue bifurcations) systems become slower in recovering from small perturbations (Wissel 1984; Strogatz 1994). A straightforward consequence of this ‘critical slowing down’ is that, if we can measure the time it takes for a system to return to its original state after a small disturbance, we may indicate the proximity of the system to a catastrophic shift (van Nes and Scheffer 2007). A range of indicators has been proposed that may reflect such slowing down in natural fluctuations or in spatial patterns (Scheffer and others 2012; Dakos and others 2015). In particular, rising temporal autocorrelation and variance have received much attention as indicators of loss of resilience before a transition. For instance, variance of phosphorus concentration in a lake is predicted to increase as conditions reach a threshold at which the lake shifts from an oligotrophic to a eutrophic state (Carpenter and Brock 2006). Similarly, temporal autocorrelation in vegetation biomass may rise before the ecosystem collapses to a desert state due to overgrazing (Dakos and others 2011). Other proposed indicators of resilience include increasing spatial correlation (Dakos and others 2010), increasing skewness (Guttal and Jayaprakash 2008), changing frequency spectra (Kleinen and others 2003), deviations in pattern formation (Rietkerk and others 2004), and truncated power law distributions (Kéfi and others 2007).

In theory, these indicators may well signal a nearby tipping point. However, detecting them in practice remains difficult. Timely and robust identification of resilience indicators requires long, high-resolution records with low measurement error that are simply unavailable in most ecological systems. Thus, it is not surprising that the best reported cases for detecting shifts come from controlled experiments in the lab where short-lived and easy-to-monitor single species populations are used (Drake and Griffen 2010; Dai and others 2012, 2013; Veraart and others 2012). The only ecological study that identified indicators of reduced resilience before a catastrophic shift in the field is a lake trophic cascade experiment that relied on the exceptional case of comparing dynamics between a manipulated and a control lake (Carpenter and others 2011).

Part of the difficulty stems from the fact that the proposed indicators are mostly indirect measures of critical slowing down. Such proxies have a number of issues that ultimately limit their potential to unequivocally detect whether critical slowing down is at play (Brock and Carpenter 2010; Dakos and others 2012, 2015). For instance, strong environmental stochasticity could muffle any rising pattern in variance caused by critical slowing down. False-positive trends in both variance and autocorrelation might be driven by changes in the pattern of environmental fluctuations rather than by the proximity to a nearby transition (Dakos and others 2015). Overall, the most reliable way to identify critical slowing down is to directly measure the time (or alternatively rate) it takes for a system to recover after a small experimental disturbance (van Nes and Scheffer 2007).

In its simplest form, one applies a *homogeneous (system-wide), weak* perturbation (for example, by removing 5% of the biomass) and measures the time it takes to recover to the pre-disturbance state. However, most ecosystems are ‘spatially extended,’ in the sense that the size of the landscape is large compared to the scale at which important processes and interactions are acting. Examples of spatially extended ecosystems with critical transitions between alternative stable states include kelp forests (Konar and Estes 2003), coral reefs (McManus and Polsenberg 2004; Elmhirst and others 2009), semi-arid vegetation (Rietkerk and van De Koppel 1997), mud-flats (van de Koppel and others 2001), and lake vegetation in large lakes (Scheffer 1998). Homogeneous (system-wide) disturbance experimentation could be problematic in practice, either due to cost and management restrictions (for example, protected habitats), or because experiments are simply impossible to perform on the scale of the entire ecosystem. For instance, one simply cannot remove a certain percentage of coral cover on an entire reef to measure its recovery. In addition, the effects of a weak disturbance might be difficult to measure in a naturally stochastic environment, while a too strong system-wide pulse experiment might ‘accidentally’ push the ecosystem to an undesirable state. Thus, such large-scale approaches do not always belong to the fail-safe experimentation that would be appropriate for measuring resilience (Holling and Meffe 1996).

In practice, it will be more feasible to perform a *strong local* perturbation, for instance by removing all vegetation in a small area in the middle of a vegetated area. This perturbation type is different from a system-wide perturbation, since spatial interactions and dispersal play a role in the recovery. Here, we study whether recovery rate from strong but local disturbances in spatially extended ecosystems can be used to infer *system*-*level* proximity to a tipping point. We show that both in continuous landscapes (for example, a single large lake or forest) and in patchy landscapes (for example, a set of connected ponds or forest patches) recovery rates upon local disturbances could reflect the proximity to a threshold for system-wide collapse. Nonetheless, we find that their performance crucially depends on the dispersal rate of organisms and the scale of perturbations.

## Methods

### Model

To test whether recovery time upon strong local perturbations can, theoretically, be used as an indicator for loss of resilience in large-scale ecosystems, we adapted a harvesting model with alternative stable states (Noy-Meir 1975) to make it spatially explicit. The basic model describes the logistic growth of a resource *N* that is harvested following a sigmoidal functional response. This model has been extensively used to study overexploitation (May 1977) and the collapse of overgrazed semi-arid vegetation (Noy-Meir 1975). For a range of parameters, resource biomass can be in two alternative states: a high biomass (underexploited) state and a low biomass (overexploited) state. Growth rate of resource *N* is given by equation 1:

1 $$ f(N) = rN\left( {1 - \frac{N}{K}} \right) - c\frac{{N^{2} }}{{N^{2} + H^{2} }} $$

in which *r* (1 day^−1^) is the local maximum growth rate of resource *N* (in g m^−2^), *K* (10 g m^−2^) is the local carrying capacity of resource *N*, *c* (ranging from 1.8–2.8 g m^−2^ day^−1^) is the maximum harvest rate, and *H* (1 g m^−2^) is the half saturation of the functional response of harvesting. An increase in harvest rate *c* (stress driver) leads to a decrease in stability of the underexploited, high biomass state and eventually pushes the system to the overexploited, low biomass equilibrium state.

**Figure 1:**
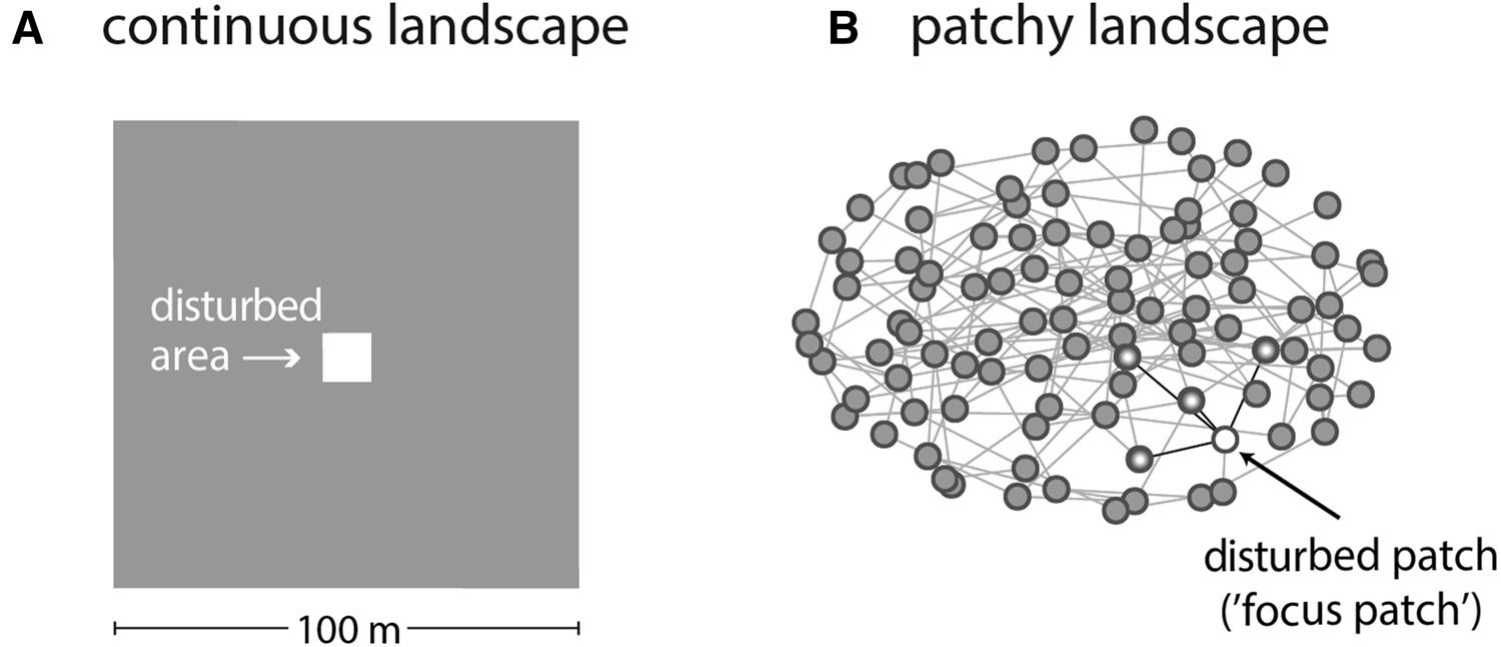
Representation of disturbance-recovery experiments in our spatially extended ecosystems. **A** A continuous landscape is defined as a fully connected landscape. We performed a local strong disturbance by removing all resource biomass from an area in the center of the landscape, indicated in *white*. **B** A patchy landscape is defined as a sparsely connected landscape: that is a network of patches that are randomly connected to other patches in the landscape. We performed a local strong disturbance by removing resource biomass from one patch, the so-called focus patch, indicated by the *white dot*. The focus patch used for the simulations is connected to four other patches, indicated by the *shaded dots.*

We considered two representative spatially extended ecosystems: a ‘continuous landscape’ and a ‘patchy landscape’ (Table 1; Figure 1). The continuous landscape was simulated using a partial differential equation (PDE) model. Dispersal of the resource through the continuous landscape was modeled in its simplest form, as diffusion with diffusion rate *D* (Table 1; Figure 1A). The patchy landscape was defined as a random network of 100 patches with 0.04 connectivity (that is, there is 4% probability that there is an edge between two patches in the network; Figure 1B). We assumed that resource biomass is well mixed within each patch, whereas dispersal occurs between connected patches with a constant rate *d* (Table 1). Qualitatively, the patchy landscape is similar to a lattice differential equation (LDE), but in our view it represents a more realistic patchy landscape than a grid. For consistency with the continuous model, we assume that all patches are of the same size (1 m^2^). The parameters *D* in the continuous landscape and *d* in the patchy landscape thus represent the level of mixing of the resource *N* across the landscape.

**Table 1:** Different Dispersal Rates and Disturbance Levels Applied in the Recovery Time Experiments for Continuous and Patchy Landscapes

	Continuous landscape	Patchy landscape
	$N^{\prime } = f(N) + D\left( {\frac{{\partial^{2} N}}{{\partial x^{2} }} + \frac{{\partial^{2} N}}{{\partial y^{2} }}} \right)$	$N_{i}^{{\prime }} = f(N_{i} ) + f_{d} \sum {(N_{j} - N_{i} )}$
Landscape size	100 x 100 m	100 patches
Low dispersal rate	*D* = 2.5 m^2^ day^−1^	*d* = 0.02 day^−1^
High dispersal rate	*D* = 12.5 m^2^ day^−1^	*d* = 0.1 day^−1^
Small disturbance (1%)	10 x 10 m	1 patch
Large disturbance (5%)	~22.4 x 22.4 m	5 patches

*A two-dimensional partial differential equation describes the dynamics of the continuous landscape, and a sparse lattice differential equation describes the dynamics of the patchy landscape. In the dispersal term of the patchy landscape, the index j refers to the patches that are connected with patch i.*

### Simulations

We started all experiments with the entire landscape in the underexploited (high resource biomass) equilibrium state. We performed a strong, local disturbance by removing all biomass either in a square in the center of the continuous landscape or in a single patch of the patchy landscape (*N*_*i*,*t*0_ = 0, where *i* denotes the local area, or patch disturbed). We compared effects for a ‘small’ versus a ‘large’ disturbed area. In the continuous landscape, a small-disturbed area was defined as an area equal to 1% of the landscape (expressed in m^2^), whereas a large disturbed area was equal to 5% (Table 1). In the patchy landscape, a small perturbation was performed on one specific focus patch (1% of the landscape). For a large disturbance, both the focus patch and its four connected patches (that is, we selected focus patches with degree 4) were set to zero biomass (5% of the landscape) (Table 1).

Usually, recovery rate is estimated by fitting an exponential model on the recovery trajectory (van Nes and Scheffer 2007; Veraart and others 2012). In our simulations, such estimation was not possible because of the non-exponential form of recovery in parts of our scenarios. Thus, we used a simpler alternative approach. We defined recovery time upon local disturbance as the time it takes (*t–t*_0_) for the resource biomass *N*_*k*,*t*_ to recover to the pre-disturbed state *N*_*k*,*t*0_ (where *k* denotes the center of the disturbed area *i*, or patch *i*). The disturbance was assumed to be recovered if the difference between *N*_*k*,*t*0_ and *N*_*k*,*t*_ was smaller than 0.1 g m^−2^. We investigated how recovery rate (defined as 1/recovery time) changes with the distance to the tipping point by changing the harvest rate *c*, using the standardized disturbances described above.

Strong local disturbances do not always recover (Keitt and others 2001; van de Leemput and others 2015). There are two other possibilities. A local strong disturbance can either trigger a systemic collapse to the overexploited state (we call this ‘induced collapse’), or the effect of the disturbance can persist, that means that it neither recovers nor triggers a systemic collapse (we call this ‘no recovery’). As recovery cannot be defined for such cases, we reported recovery rates only when there was actual recovery. Overall, we estimated recovery rate upon local disturbances for different scenarios that are summarized in Table 1: at low and high dispersal rates, and for small and large disturbed areas.

In the baseline scenarios, we assumed a homogenous landscape (that is, the same parameters across space) and standardized disturbances (that is, same size and location of the disturbed area). In addition, we performed experiments in the presence of spatial heterogeneity, with randomly located disturbances. We introduced spatial heterogeneity in both landscapes by varying the maximal growth rate of the resource in space (*r*). To create heterogeneity in the continuous landscape, we first split the landscape in 25 equally sized squares and randomly assigned growth rates from a uniform distribution (*r* ~ *U*[0.8, 1.2 day^−1^]). Next, we smoothened the generated variability over the entire space with a Gaussian smoothing function (Bowman and Azzalini 1997). In the patchy landscape, growth rates were randomly assigned to patches following the same uniform distribution (*r* ~ *U*[0.8, 1.2 day^−1^]).

Randomly located disturbances were introduced as follows. In the continuous landscape, the size of each disturbance (as percentage of the total area) was drawn from a uniform distribution (size ~ *U*[0.01, 0.05], whereas the location of the disturbance was determined randomly in the landscape. In the patchy landscape, we selected one patch at random for each simulation (patch ~ *U*[1,100]). Due to the network topology, the disturbed patches differ in their number of neighbors (that is, degree). We define a single ‘recovery rate experiment’ as a collection of simulations with one random disturbance per simulated level of harvest rate *c.* To get an estimate of the variability in recovery rate, we simulated 100 recovery rate experiments. We reported the mean recovery rate and the 10th and 90th percentiles for each level of the harvest rate *c*. Moreover, we reported the percentage of simulations that yield ‘no recovery,’ and we reported indicators after correcting for the size of disturbance *A* (in m^2^) in a continuous landscape, and the degree of the disturbed node *k* in a patchy landscape. We did this by regressing recovery rate $\frac{1}{\Delta t}$ against perturbation size *A* in the case of a continuous landscape and degree of perturbed node *k* in the case of a patchy landscape, to get an estimated recovery rate for each perturbation experiment$\left( {\frac{{\hat{1}}}{\Delta t}} \right)$. We reported residuals $\left( {\frac{1}{\Delta t} - \frac{{\hat{1}}}{\Delta t}} \right)$ and their 10th and 90th percentiles.

For all analyses, we used GRIND for MATLAB (accessed at http://www.sparcs-center.org/grind). We approximated a continuous landscape using the finite difference method where we discretized space in a lattice, while making sure that cells were sufficiently small to approximate continuous space for the parameters we used. This resulted in a 50 × 50 lattice. Importantly, a finer-meshed lattice did not alter the resulting dynamics for the parameters we used. Note that one should always be aware of situations of no recovery due to the discretization method used (Keitt and others 2001; van de Leemput and others 2015). All differential equations were solved using an explicit Runge–Kutta (4, 5) solver with adaptive step size.

## Results

**Figure 2:**
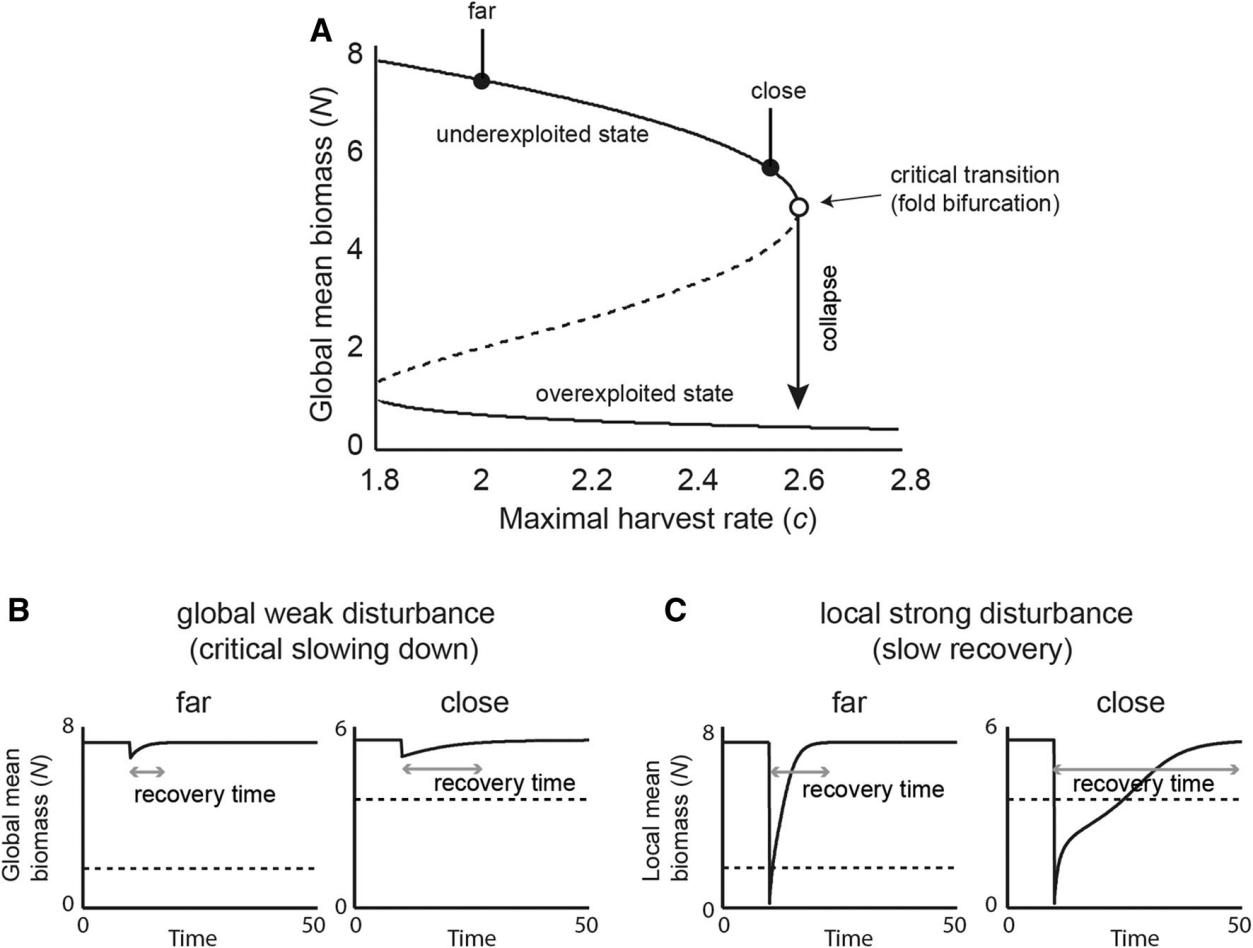
Collapse of resource biomass under increasing harvesting, and two distinct disturbance-recovery experiments in a spatially continuous ecosystem. **A** Bifurcation diagram of resource biomass. Increasing harvest rate *c* pushes the resource (mean biomass) toward the tipping point to overexploitation. Continuous lines indicate the two alternative equilibria. The dashed line indicates the unstable equilibrium that divides the two alternative basins of attraction. **B** We performed a weak global disturbance (by removing 10% of standing biomass) far (*c* = 2) and close (*c* = 2.55) to the transition and monitored an increase in recovery time due to critical slowing down. **C** We performed a strong local disturbance (by removing all standing biomass in an area comprising 1% of the landscape) far (*c* = 2) and close (*c* = 2.55) to the transition and monitored an increase in recovery time. Note this increase is not strictly due to critical slowing down. Dashed horizontal lines indicate the threshold between the basins of attraction of the two alternative states. For all simulations, dispersal rate is low (*D* = 2.5 m^2^ day^−1^).

In both our spatial systems (Figure 1), increasing the harvest rate caused a gradual decrease in resource biomass up till the tipping point at which the ecosystem collapsed to the alternative overexploited state (the fold bifurcation point in Figure 2A). In line with previous results (van Nes and Scheffer 2007), the time of the system to recover upon a weak global perturbation becomes longer as the system is closer to the bifurcation point (Figure 2B).

Slowing down was also observed in the time to recover from resource biomass removal in a small area (Figure 2C). Note, though, that the recovery trajectory of a strong local perturbation can be non-exponential (Figure 2C). Close to the systemic tipping point, the total biomass on the landscape may even decrease prior to recovery (see Online Appendix 1). This can happen if the conditions are such that the system has already crossed the Maxwell point: The basin of attraction of the low biomass state is larger than the basin of attraction of the high biomass state. Under these conditions, there is potential for a sufficiently large local perturbation to induce a traveling front toward systemic collapse (van de Leemput and others 2015). The reason we do not see the full collapse in this example is that the perturbation was too small to actually induce such collapse and the system eventually recovered. The decrease in resilience of the high biomass state was reflected in the local recovery time (Figure 2C).

In addition, the size of the area that was affected by the perturbation could be considered an indicator of resilience (see also Dai and others 2013 for a similar measure of recovery length). Systematic analysis of the size of the affected area suggested that it might be an appropriate metric that rises steadily as the system approaches critical conditions for systemic collapse (see Online Appendix 1). In what follows, we limit our further analysis to recovery rate of the perturbed site itself.

**Figure 3:**
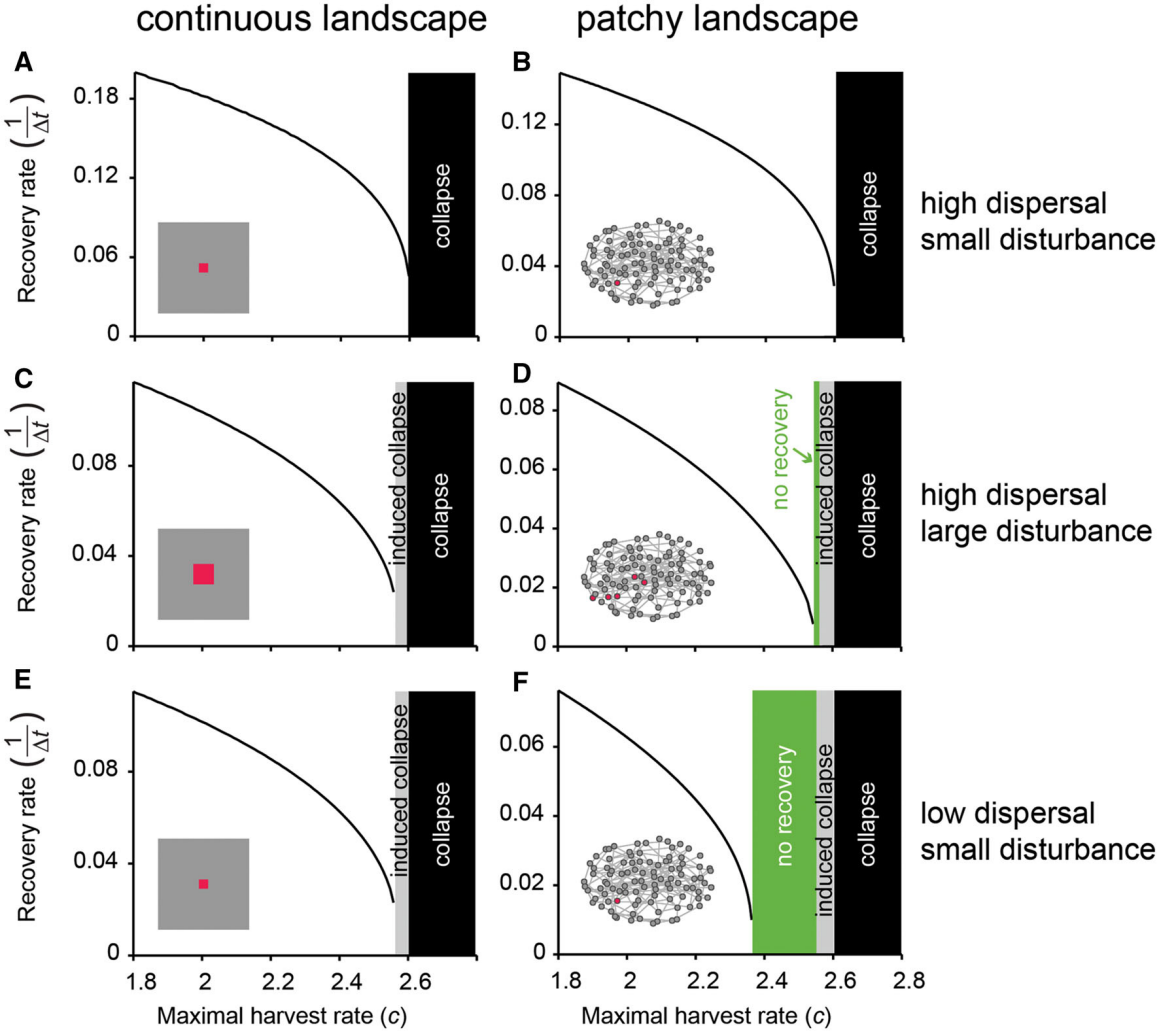
Strong local disturbance-recovery experiments for measuring recovery rate (as 1/recovery time) as an indicator for ecosystem-level resilience. **A** Recovery rate upon a local disturbance—a zero-biomass area in the middle of a homogeneous high-biomass landscape (indicated by the *red area*)—as a function of harvest rate *c*. **B** Recovery rate upon a local disturbance—a zero-biomass patch in a patchy high-biomass landscape (indicated by the *red focus patch*)—as a function of harvest rate *c*. **C**, **E** In a continuous landscape, a large disturbance in a system with a high dispersal rate or a small disturbance in a system with a low dispersal rate may induce a ‘premature’ systemic collapse (*gray area*). This means that a transition of the global ecosystem takes place before the actual fold bifurcation point (*black area*). **D**, **F** In a patchy landscape, a local disturbance may also induce a systemic collapse (*gray area*), but can also lead to no recovery (*green area*) especially when the dispersal rate is low.

In general, we found that recovery rate (that is, 1/recovery time) decreased smoothly as increased harvest rates brought the system closer to the tipping point for a systemic collapse. This was true in both continuous and patchy landscapes (Figure 3A, B). As the perturbed area was larger, it invoked a systemic collapse at lower harvest rates (‘induced collapse’) (Figure 3A vs. C and B vs. D).

The overall difference between a homogeneous continuous (Figure 3A, C, E) and a patchy (Figure 3B, D, F) landscape is that a local disturbance in a continuous landscape will always either recover or expand (‘induced collapse’), whereas in a patchy landscape there is a range of conditions at which a local disturbance can persist in time (‘no recovery’), neither recovering nor expanding (see also van de Leemput and others 2015).

Results also depended on the dispersal rate of the resource. In a system with a low dispersal rate, even small-scale perturbations could provoke premature systemic collapses in the continuous landscape (Figure 3E). In a patchy landscape, the ‘no recovery’ range was larger in a system with a low dispersal rate (Figure 3F), compared to a system with a high dispersal rate (Figure 3D). Not surprisingly, when we imposed a large disturbed area on a system with a low dispersal rate, the probability of a premature collapse or no recovery increased even more (Online Appendix 2).

In spite of those differences, recovery rates always decreased as the harvest rates approached the critical point where a premature collapse or ‘no recovery’ situation occurred (Figure 3C, D, E, F). Thus, rather than the distance to the generic bifurcation point, a drop in recovery rates signaled the decreased capacity of the ecosystem to recover from the prescribed local perturbations.

**Figure 4:**
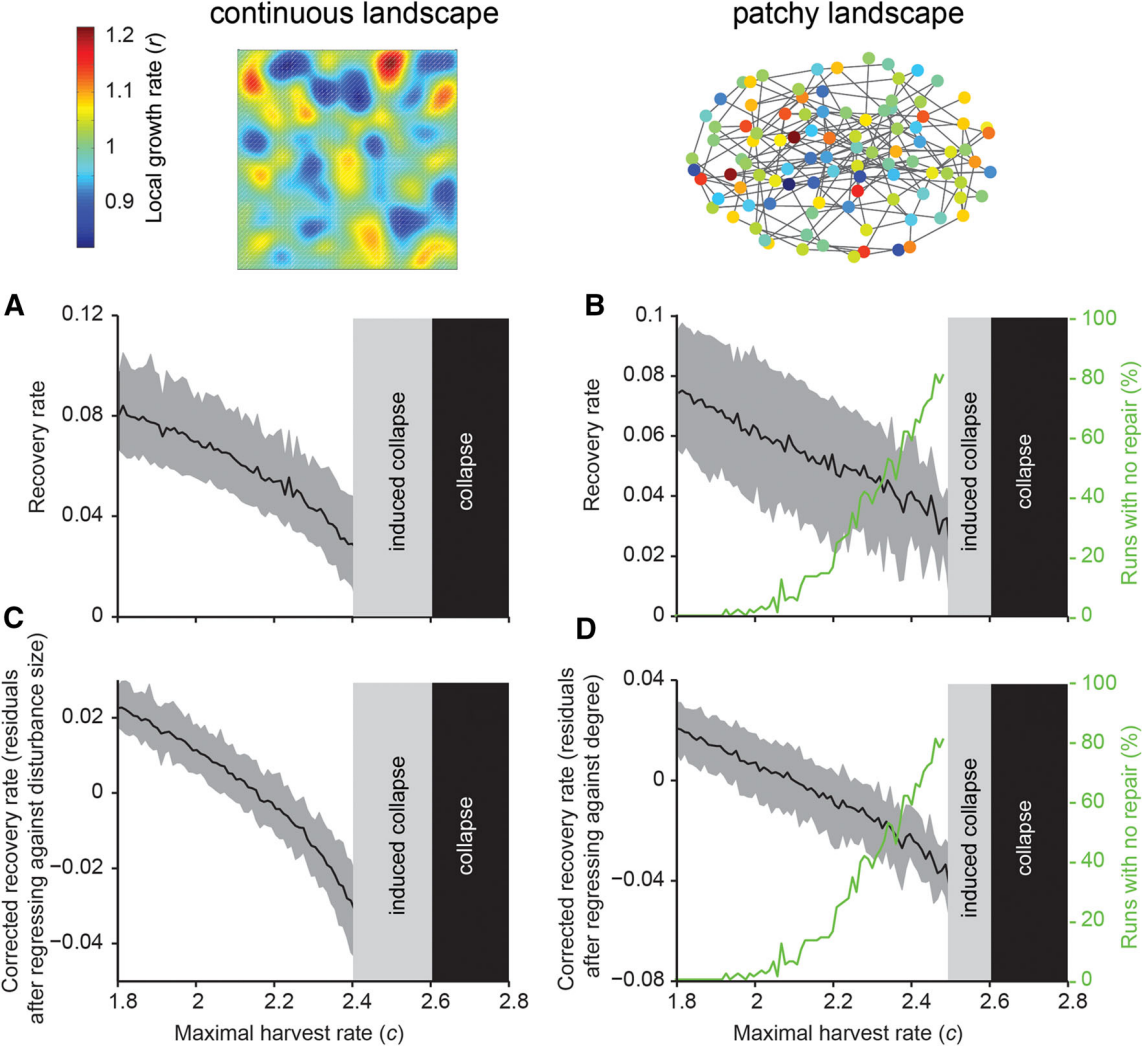
Effects of random disturbance experiments in landscapes with spatially heterogeneous conditions and a low dispersal rate. We randomly disturbed areas of different size on a continuous landscape and different patches in a patchy landscape for each level of resilience (in terms of maximal harvest rate) and measured recovery rates. **A**, **B** Average recovery rates decrease in all situations as the system approaches the critical harvest rate for collapse. *Black lines* represent the average recovery rate, while the *gray shaded areas* show the 10th and 90th percentile, based on experiments per simulated harvest rate. **B** In a patchy landscape, the percentage of experiments followed locally by no recovery (*green lines*) increases as the harvest rate increases. This occurs especially when the dispersal rate is low (see Appendix 2 for a system with a high dispersal rate). **C**, **D** Average residual recovery rates after being corrected for the size of the disturbance (**C**), or the degree of the disturbed node (**D**). Note that the variance in recovery rates is lower after the correction.

So far, we assumed that environmental conditions across the landscape were homogeneous. As a next step, we considered a situation where conditions (represented by the maximum growth rate *r*, see methods) are varied spatially (Figure 4). To analyze the properties of such heterogeneous landscapes, we performed multiple experiments in which we simulated disturbances of random size at random locations. There was a clear decreasing trend in such average recovery rates as increasing harvest brought the landscapes closer to the systemic collapse (Figure 4, see Online Appendix 2 for a system with high dispersal rates). Still, variability was relatively large, which was due to 1) the location of the disturbance in the heterogeneous landscape and 2a) the variation in size of disturbance in a continuous landscape, or 2b) number of neighbors (degree) of the disturbed node in the patchy landscape. Importantly, if data were available on any of these variables, one might be able to reduce the variability in recovery rates (Figure 4C, D; Online Appendix 2), and improve the sensitivity of the resilience indicator. In the patchy landscape, the probability of local no recovery upon perturbations strongly increased toward the systemic collapse (Figure 4B), which was much less when dispersal rates were high (Online Appendix 2).

## Discussion

Our analyses suggest that in spatially extended ecosystems, reduced recovery rates upon local perturbations may signal that the ecosystem is approaching a system-wide transition. At the same time, our results show that local recovery rates depend strongly on the size of the local perturbations and on the exchange of resources between the perturbed area and its surrounding. The level of exchange essentially depends on the dispersal rate and in a patchy landscape on the connectivity (that is, degree) of the disturbed patch. Although these findings may seem straightforward at first sight, the link to practical implications as well as the more fundamental underlying theory is not so clear.

### Signals from Strong Local Disturbance Versus Weak Global Disturbance Experiments

Although our results resemble the patterns found by measuring recovering rate upon a small perturbation in a well-mixed system (van Nes and Scheffer 2007; Dakos and others 2011), they are not directly related to the same phenomenon of critical slowing down. Critical slowing down is defined for weak disturbances close to equilibrium (Figure 2B) where recovery is approximately exponential. In our strong local disturbance experiments, we pushed a local area to the alternative equilibrium (Figure 2C). As the disturbed area lies in the basin of attraction of the alternative state, it would not recover in case it was isolated. Inflow of biomass from the neighboring undisturbed parts of the system is needed. The capacity for this recovery process depends on the exchange between the perturbed area and its surrounding (that is, dispersal rate), but also on the conditions that determine local resilience. Phrased loosely, a system weakened by harsh conditions (that is, here represented by a high harvest rate) will recover more slowly from local damage, because the neighboring area has a low capacity to ‘pull’ the disturbed area back to the pre-disturbed state. In theoretical terms, the change in the relative resilience and reduction of the recovery capacity of spatially extended systems is related to the crossing of a Maxwell point (Keitt and others 2001; Bel and others 2012). At this theoretical point, in parameter space both equilibria are equally stable (that is, they have the same potential (Strogatz 1994)) and large spatial perturbations will neither recover nor expand (van de Leemput and others 2015).

Beyond the theoretical aspects, our results have marked practical implications. They suggest that a local-scale experiment can provide information for the resilience of a large-scale ecosystem. This is important as performing a small-scale disturbance experiment is much more realistic than applying a large-scale disturbance. For example, instead of facing the daunting task of removing 10% of submerged vegetation in a whole shallow lake, one may probe the resilience of the lake by removing all vegetation from just a small area. In fact, the detectability of such local recovery rates might be stronger when compared to recovery rates from weak global perturbations under noisy conditions (Online Appendix 3). Also, one only needs to monitor recovery of a small part of the landscape. It should be noted that these implications apply specifically to spatially extended systems (that is, low level of mixing in relation to the size of the landscape). In case of a well-mixed system, where local perturbations smoothen out rapidly over the entire landscape, it would be more valuable to monitor the population size of the entire landscape.

Our results also suggest that detecting altered local recovery rate may be feasible for random disturbances in a heterogeneous environment (Figure 4). Obviously, this approach still requires having sufficient replicates of the disturbance experiments to get accurate results. Importantly, one can reduce some of the observed variability in recovery rates if one has relevant information on the disturbances (Figure 4C, D). For instance, one might be able to correct for the size of the disturbances (Figure 4C). Also, if connections between nodes in a patchy landscape are known, one could correct for the degree of the disturbed node (Figure 4D), because the connectivity of a patch largely determines the exchange rate between the disturbed node and its surrounding.

### Designing Experiments

Clearly, our models are quite abstract, and bridging from our results to any particular field situation is a challenge. Nonetheless, our results suggest some aspects to ponder when it comes to designing experiments. First of all, disturbance experiments are never completely free of risk. The local disturbance may unintentionally trigger a domino effect, such that the disturbance spreads through the entire landscape (Peters and others 2007). The likelihood of such an induced system-wide collapse depends on the spatial extent of the perturbation, the overall ecosystem resilience (that is, the level of the stress driver), and the strength of dispersal (see Online Appendix 4). It also depends on the type of connectivity in the landscape, that is, whether the landscape is continuous or patchy (Figure 1). Even a very local perturbation can theoretically trigger a collapse in a continuous landscape that is close to a tipping point, provided that the dispersal rate is low (Figure 3E). This is because at low dispersal rates, the capacity of the landscape at larger scales to recover from a local disturbance is reduced. As a result, the local effect may persist long enough to kick-off a domino effect leading to an expanding collapse (van de Leemput and others 2015).

Obviously, spatial interactions that are relevant to recovery are typically more complex than the simple diffusion mechanism in the model. Plants may enter through seed dispersal or root expansion depending on species and conditions, and animals may move directionally in or out of damaged patches. For example, clearing part of seagrass meadows creates open spaces attracting swans that delay or prohibit the regrowth of seagrass (van der Heide and others 2012). Also, other processes may play a role in the recovery of local disturbances. For example, regrowth of vegetation patches may not occur from the side of a cleared patch, but simply from overwintering structures belowground.

In general, there is a trade-off between signal strength and the probability of inducing a collapse. Researchers in any particular ecosystem probably have a good intuition about the type and size of experimental perturbation that could yield a clear recovery signal, while not posing a risk of inducing a spreading collapse. We summarize some of the opportunities and limitations of recovery of local disturbances as an indicator of resilience in Table 2.

**Table 2:** Opportunities and Limitations for Performing Local Disturbance-Recovery Experiments in Spatially Extended Ecosystems to Indicate System-Wide Resilience

Local recovery time experiments	
opportunities	limitations
Are feasible to perform	Can accidentally induce a collapse
Are easy to monitor	Might not reflect system-level resilience (under extreme landscape heterogeneity)
Provide a strong signal even under stochastic conditions	Require multiple experiments for averaging out local differences
Natural disturbances can be used as proxy experiments	Require information on dispersal rates and landscape heterogeneity Variability in dispersal rates and perturbation size can muffle the effect of resilience
Can be performed at different spatial scales	May initiate other processes that lead to alternative outcomes

Although replicated prescribed experiments are the cleanest way to monitor change in recovery rates, natural local disturbances may offer an alternative in some situations. Recovery from events such as disease outbreaks, wildfires, or bleaching has been studied in ecosystems ranging from grasslands (Tilman and Downing 1994), and marine kelp ecosystems (Dayton and others 1992) to forests (Cole and others 2014), and coral reefs (Houk and others 2014). Especially if data on many repeated events are available, differences in recovery rates may hint at differences in resilience. This should work best in a mostly homogeneous environment.

### Restoration: A Flipped Perspective

In our modeled patchy landscapes, there is the possibility that a disturbed patch does not recover but that there is no cascading effect causing the entire landscape to shift to the other state [that is, it has an infinite recovery time, the so-called pinning (Keitt and others 2001; van de Leemput and others 2015)]. In practice, partial transitions may be more common than all-or-none transitions, as even modest heterogeneity in conditions or in dispersal rates can allow spatial coexistence of alternative stable states (van de Leemput and others 2015). How one looks at such transitions depends on the context. Clearly, the words ‘collapse’ and ‘recovery’ we use are value-laden, suggesting that the current state of the landscape is preferred over the potential alternative state. However, one may flip the perspective and frame our results thinking of the transition to the alternative state as a ‘restoration’ that brings the system to a preferred state. For instance, one may try to eradicate an invasive species, or promote the return of vegetation that originally dominated the landscape. In such situations, the ‘no recovery’ results may correspond to successful restorations of parts of the landscape. A cascading ‘collapse’ would be a large-scale success, and ‘recovery’ would be a failure. The same results then illustrate that depending on the tendency of species to spread (that is, our dispersal rate) a sufficiently large scale of restoration efforts can be critically important, especially in homogeneous landscapes (van de Leemput and others 2015). Importantly, the interpretation of ‘recovery rate’ (that is, rate at which the system returns to the original state after a restoration attempt) as an indicator of resilience remains relevant, as it may be used to probe whether a system may easily be restored or not. Areas with the lowest recovery rates may in that perspective be the most promising places for restoration efforts, and the effect of the perturbed initial patch on recovery rates may give an indication of the critical scale needed for successful restoration.

### Prospects

The analysis of recovery rates in spatially extended ecosystems is an almost unexplored territory (Dai and others 2013; Benedetti-Cecchi and others 2015). Our results suggest a number of ways forward. It is easy to see the scope for prescribed replicated experiments along environmental gradients where the system is known to approach a critical transition. On small scales, such as laboratory systems or herbaceous vegetation, this may be quite feasible. For instance, strikingly clean results can be found in elegant experiments with yeast populations growing in a set of flasks where spatial interactions are simulated by manually dispersing yeast between flask cultures on a daily basis (Dai and others 2013). On larger scales, it may be possible to interpret the response to frequent human-induced or natural perturbations. For instance, remotely sensed recovery of tropical forests from wildfires or clearing could indicate spatial variation or long-term trends in resilience.

Also, on the theoretical side there is scope for further exploration. We have only briefly touched upon the transient spatial expansion of a disturbance as an indicator of resilience. A related suggestion has been done for the slightly different situation of a press perturbation (for example, a continuous local harvest of biomass instead of a one-time removal). Here recovery time is no relevant measure, as the system is not allowed to recover. Instead, the size of the area impacted by the disturbed region, termed ‘recovery length’, indicates the resilience of the system (Dai and others 2013). Furthermore, we did not consider the effect of network topology here. If one considers the landscape as a network of habitat patches, future work could explore which patches, under which conditions, could be used best as target nodes to indicate systemic resilience in disturbance-recovery experiments.

Analyses of such spatial aspects complement the existing body of work on indicators of critical slowing down in simple time series. Clearly, the combination of spatial and temporal dimensions of the response to local perturbations contains most information. The two are not redundant, as the extent of spatial expansion carries information on spatial processes that cannot be inferred from the local response alone. Thus, their combination may allow separating the relative importance of intrinsic dynamics and spatial interactions. Developing and testing indicators based on spatiotemporal responses is an exciting and promising way forward.

## Electronic supplementary material

Below is the link to the electronic supplementary material.

Supplementary material 1 (DOCX 742 kb)
