# Supplementary material for: Slow Recovery from Local Disturbances as an Indicator for Loss of Ecosystem Resilience
Source: Ecosystems. 2017 Jun 2;21(1):141–52. doi: 10.1007/s10021-017-0154-8 (PMC6954009; doi:10.1007/s10021-017-0154-8)
Supplement: Supplementary file 1 — Supplementary material 1 (DOCX 742 kb) [file 10021_2017_154_MOESM1_ESM.docx]

**Appendices**

***Online Appendix 1: Spatial extent of local strong disturbances in continuous landscapes as indicator of resilience***

Our model simulations show that the recovery trajectory of a local perturbation in a continuous landscape can under certain conditions (i.e. low resilience and low dispersal rate) comprise several phases of recovery (see for example Fig. 2C in the main text). Such recovery trajectories can be explained by an initial spatial expansion of the disturbance (see Movies A1). Figure A1 A and B show the landscape-wide recovery trajectories for the same simulation in Fig 3C. Figure A1 B shows that the total biomass on the landscape decreases after the perturbation, but recovers eventually.

We also calculated the size of the affected area of a local strong perturbation. This alternative measure is related to the concept of ‘recovery length’ which has been introduced as an indicator of system resilience by Dai and others (2013). While the authors use a local *press perturbation*, we calculate the extent of spatial expansion of a local *pulse perturbation*. Our aim is to map the spatial expansion of a disturbance as the system is approaching a critical transition. For the calculations, we used a one-dimensional version of the model described in the main text (with *L*=100 m, low dispersal rate *D*=2.5 m^2^day^-1^, and high dispersal rate *D*=12.5 m^2^day^-1^, and a disturbance size of 5% of the landscape (=5 m)). As a first measure, we monitored the size of the affected area at a specific time after the disturbance (*Δt*), The affected area is defined as the size of the area in which local biomass is below 90% of the biomass before the disturbance.

Our results show that the longer the time interval, the less detectable the recovery length far from the transition (Figure A2 A, C). For instance at *Δt* = 20 days*,* changes in recovery length are detectable after harvest rate has crossed 2.4 g m^-2^ day^-1^, which is close to the area of induced collapse (red area Figure A2 A, C). On the other hand, for short time intervals of *Δt* = 5 days, recovery length is detectable far from the transition, however the differences between the extent of spatial expansion are small for different harvest rates. It is only at intermediate time intervals where changes in recovery length can be used as a detectable indicator. If that optimal time interval is known, the practical advantage of measuring spatial expansion at a specific time after the disturbance is that one has to visit the disturbed area only once. As a second measure, we determined the maximum size of the affected area during the recovery phase (Figure A2 B, D). This shows to be a good alternative for recovery rate as an indicator of system-wide resilience, especially if the dispersal rate is low (Figure A2 B). However, in practice, similar to recovery rate, one needs to continuously monitor the entire recovery phase to get a good measure. The maximal spatial extent can however be a good indicator in ecosystems for which biomass decrease leaves a well-detectable trace (e.g. waste product).

**Movies A1: https://www.dropbox.com/sh/bubrdemy22o8blw/AACy1HIgvlW9CYHyfdMut3cRa?dl=0**

**
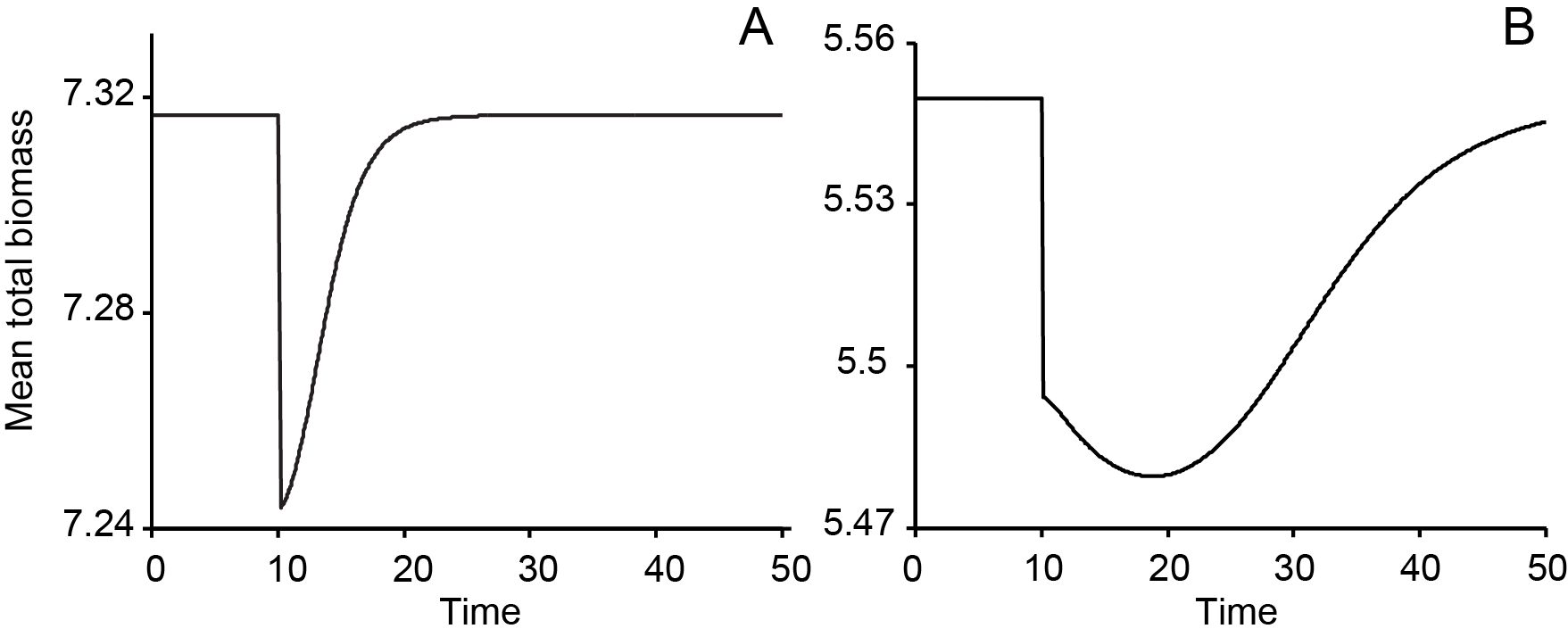
**

**Figure A1.** Landscape-wide recovery trajectories of a strong local disturbance by removing all standing biomass in an area comprising 1% of the landscape **(A)** far (*c*=2.0), and **(B)** close (*c*=2.55) to the transition in a continuous landscape with a low dispersal rate for the modelled resource (*D*=2.5 m^2^ day^-1^).


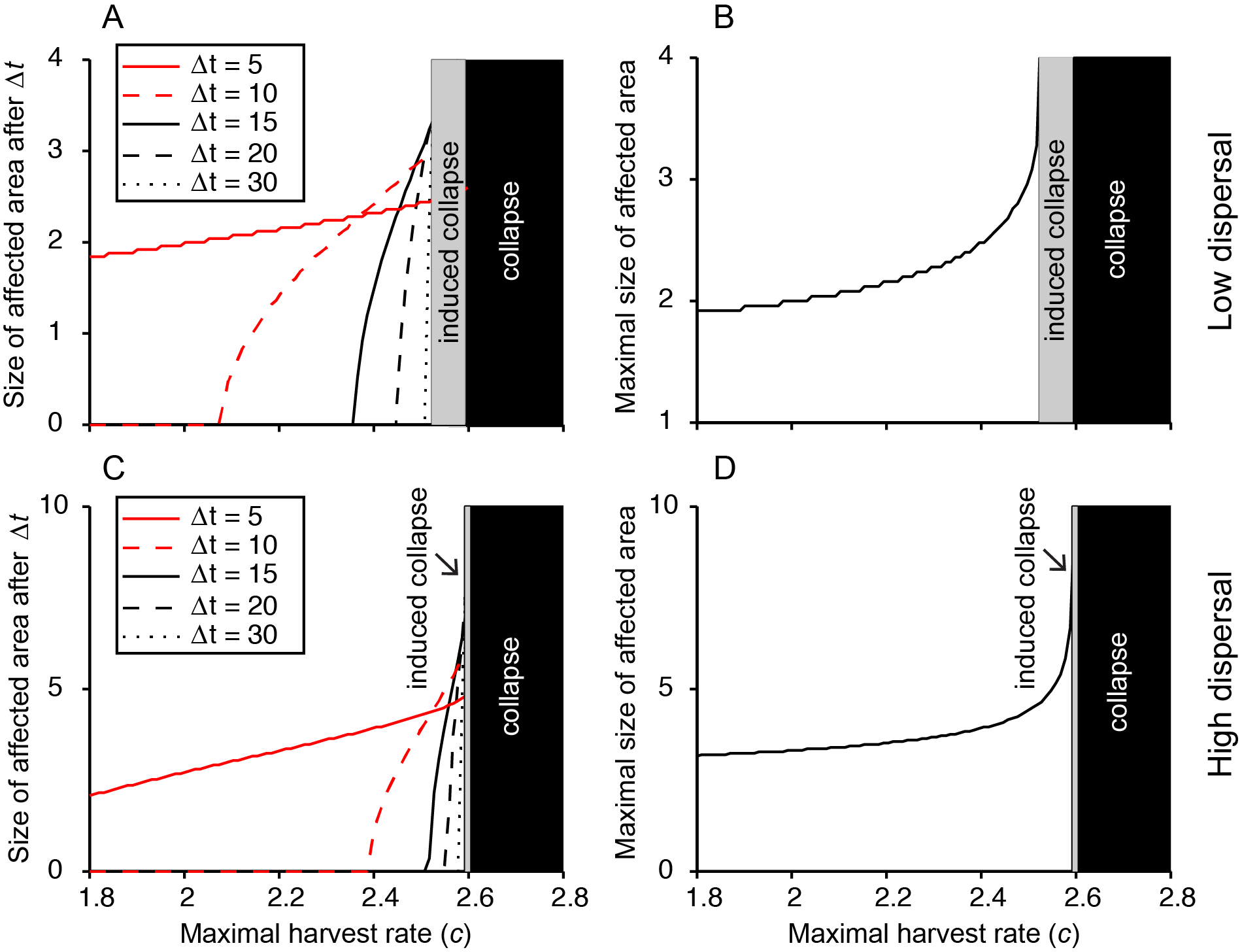


**Figure A2.** The size of the affected area after a local perturbation of 5 m, in a one-dimensional continuous landscape. If local biomass is below 90% of the biomass before the disturbance, the location is defined ‘affected’. The left panels show the size of the affected area at a single point in time after the disturbance. The right panels show the maximal size of the affected area during the period after the disturbance. (**A and B)** Low dispersal rate (*D*=2.5 m^2^ day^-1^), **(C and D)** High dispersal rate (*D*=12.5 m^2^ day^-1^).

***Online Appendix 2: Recovery rate of local disturbances***

We experimented with large disturbances in continuous and patchy landscapes where the resource has a low dispersal rate. We induced a disturbance comprising 5% of the landscape with a dispersal rate of 2.5 m^2^ day^-1^ in the continuous landscape (Figure A3 A), and a dispersal rate between nodes of 0.02 day^-1^ in the patchy landscape (Figure A3 B). Under these conditions the chances for inducing a collapse or having no recovery after a disturbance are high. Indeed, we found that the range of induced collapse in the continuous landscape considerably increased when compared to conditions of a high dispersal rate but a small-scale disturbance (Figure 3C main text), or a small-scale disturbance but a low dispersal rate (Figure 3E main text) (Figure A3 A). Similarly, the range of induced collapse and no recovery in the patchy landscape considerably increased when compared to conditions of a high dispersal rate but a small-scale disturbance (Figure 3D main text), or a small-scale disturbance but a low dispersal rate (Figure 3F main text) (Figure A3 B).

We also experimented with local disturbance-recovery experiments in a heterogeneous landscape with variable size of the disturbed area and a high dispersal rate (Fig. A4). All parameters and simulations are the same as for Figure 4 in the main text, only the dispersal rate is high.


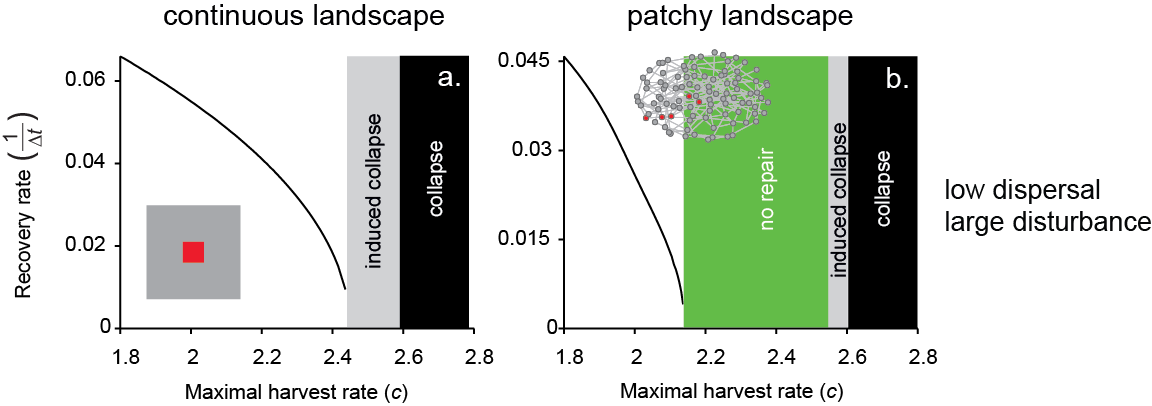


**Figure A3.** Recovery rate (as 1/ recovery time) upon a large (5% of the landscape) local disturbance -a zero-biomass area in the middle of a homogeneous high-biomass landscape (indicated in red) as a function of harvest rate *c* up to the crossing of the critical transition in **(A)** a continuous landscape with a low dispersal rate (*D*=2.5 m^2^ day^-1^), and **(B)** a patchy landscape with a low dispersal rate between patches (*d*=0.02 day^-1^). The disturbed area in (A) and the disturbed patches in (B) are indicated in white. Note that in a continuous landscape (A), the large disturbance can induce a systemic collapse (grey area) far before the actual fold bifurcation point (black area). In a patchy landscape, the large disturbance may also induce a systemic collapse (grey area), but far before that point the disturbance leads to no recovery, i.e. partial collapse (green area).

**
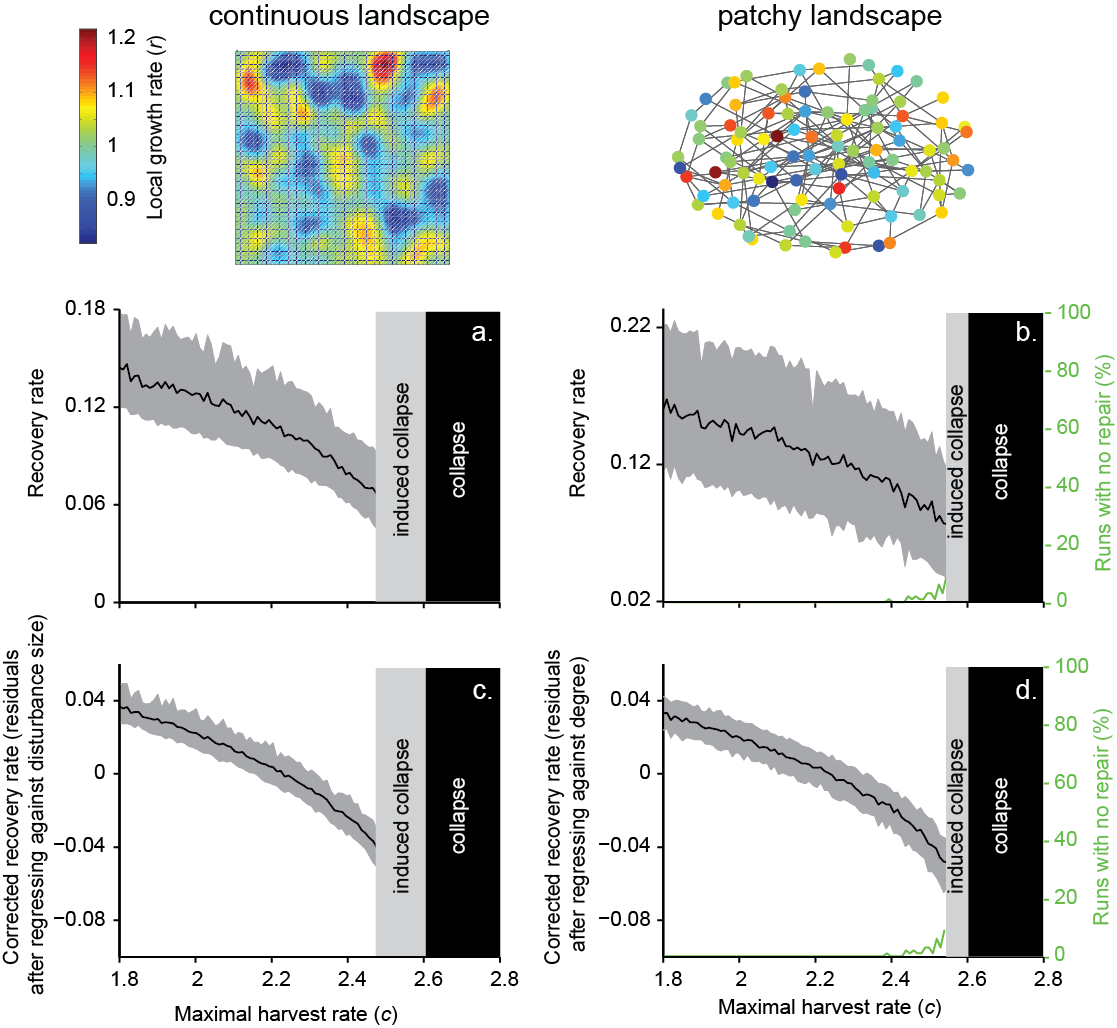
**

**Figure A4.** Effects of random disturbance experiments in landscapes with spatially heterogeneous conditions, and a high dispersal rate. We randomly disturbed areas of different size on a continuous landscape and different patches in a patchy landscape for each level of resilience (in terms of maximal harvest rate) and measured recovery rates. (**A**-**B**) Average recovery rates decrease in all situations as the system approaches the critical harvest rate for collapse. Black lines represent the average recovery rate, while the grey shaded lines show the 10^th^ and 90^th^ percentile, based on experiments per simulated harvest rate. (**B**) In a patchy landscape, a small percentage of simulations show no recovery (green lines), which increases as the harvest rate increases. (**C**-**D**) Average residual recovery rates after being corrected for the size of the disturbance (C), or the degree of the disturbed node (D). Note that the variance in recovery rates is lower after the correction.

***Online Appendix 3: Recovery time after strong local disturbances in the presence of environmental stochasticity***

We compared recovery time after a global weak disturbance to recovery time after a local strong disturbance in the presence of environmental stochasticity. We did this by adding environmental noise to the continuous landscape as a random normally distributed variable *E*~*N*(0,1) with standard deviation *σ* (0.8) applied every *Δt* (0.1 days) timesteps (). Figure A5 (panels A, C) show that it is difficult to distinguish measurable changes in recovery time after a global but weak disturbance. Instead, a local strong disturbance is more easily distinguished from background stochasticity (Figure A5 B, D).


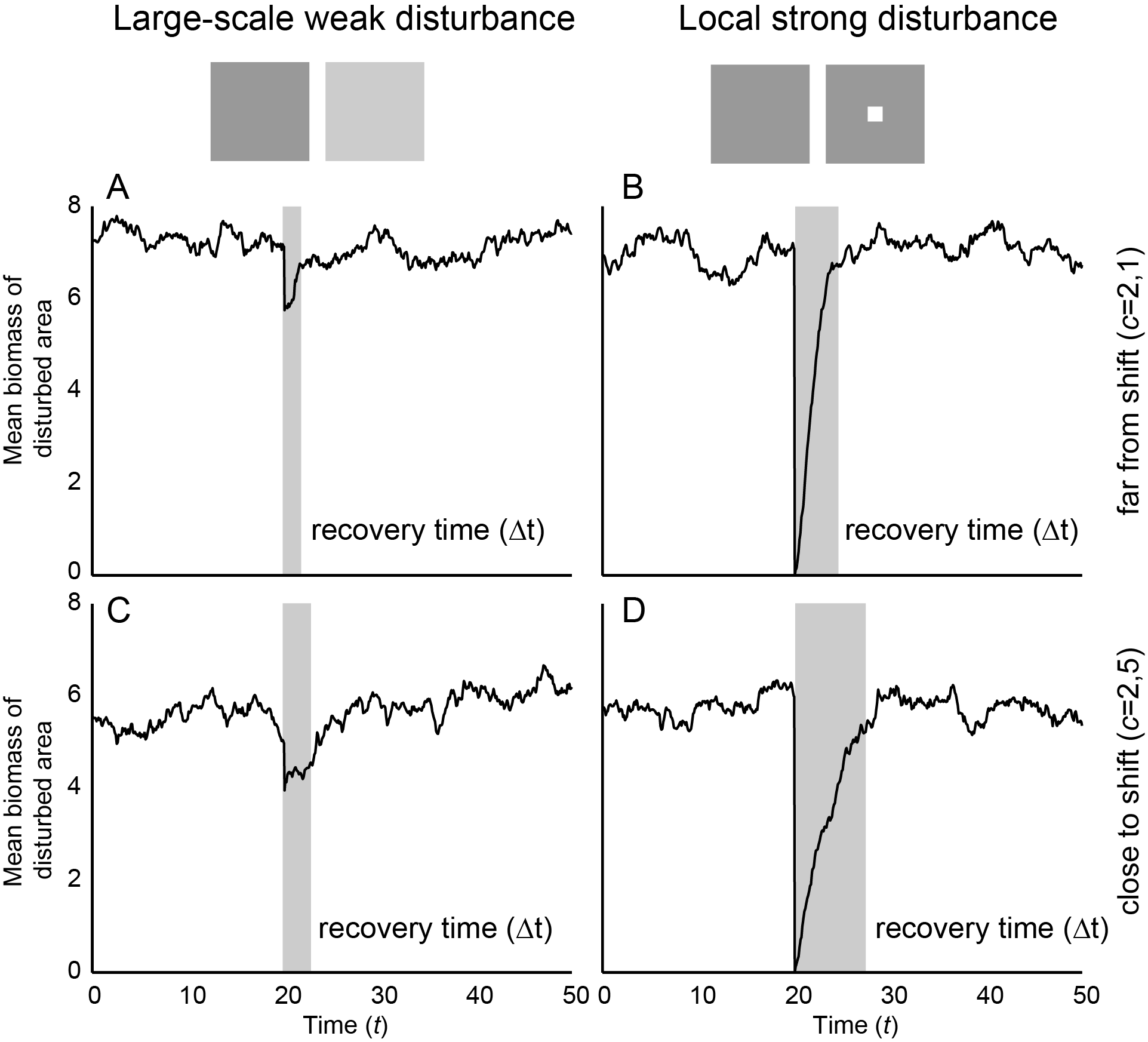


**Figure A5.** The effect of random fluctuations on system-wide and local disturbance-recovery experiments in a continuous landscape **(A and B)** far from the fold bifurcation, and **(C and D)** close to the fold bifurcation. Stochasticity in a homogeneous landscape may muffle the accuracy of the signal of a large-scale weak perturbation (A and C), while a local, strong perturbation can more easily be distinguished from background noise (B and D). For all simulations, we removed 2.5% percent of the total biomass on the landscape, and the dispersal rate was assumed to be high (*D*=12.5 m^2­^).

***Online Appendix 4: The role of dispersal rate, resilience and disturbance size on ‘induced collapse’ and ‘no recovery’ in spatially extended ecosystems***

We explored how the three responses after a strong local disturbance we presented in the main text are affected by the dispersal rate of the resource, the resilience and the disturbance size. For this, we simulated the overharvesting model on a square lattice of discrete patches where each patch was connected to its 4 neighbors. We did this as this version is a hybrid between the continuous and the patchy landscape and summarizes the dynamics of both landscape types. We assumed that all parameters were equal in all patches. Dispersal was defined as a local exchange between patches. We used a lattice with 50x50 patches.

$${N_{i,j}}^{'}=f\left( N \right)+d\left( N_{i+1,j}+N_{i-1,j}-2N_{i,j} \right)+d(N_{i,j-1}+N_{i,j+1}-2N_{i,j})$$

We defined the three different model behaviors that we observed as presented in the main text: (1) recovery: the disturbed area returns to the pre-disturbed state, (2) no recovery: the disturbed area remains stable, and (3) induced collapse: the disturbed area expands gradually, pushing the whole system eventually to the alternative stable state. We mapped all different behaviors for a set of 25x100 parameter combinations of dispersal rate *d*, resilience (harvest rate *c*), and size of disturbance. The presented 2D bifurcation plots were sketched manually on the basis of the simulation results using an overlay graph.

We first checked which of these behaviors occur at different harvest rates and dispersal rates (Figure A6 A). The harvest rate affects the resilience (*sensu* Holling 1973) of the undisturbed state compared to the state in the disturbed patch. At high harvest rates it becomes more likely that a global shift to the perturbed state occurs (“induced collapse”), but there is no single critical harvest rate above which this always happens. More interesting is the effect of the dispersal rate. If the dispersal rate is low, it becomes likely that the system neither recovers nor collapses as a consequence of the disturbance. This is because the landscape becomes similar to a patchy landscape (i.e. connectivity between cells is low): the exchange between the disturbed area and the neighboring undisturbed area is insufficient to cause a shift of either of them to the other state. As illustrated by our analysis, it is the interplay of resilience (in our case determined by the harvest rate) and the dispersal rate that determines what will happen. At intermediate harvest rates, the probability of no recovery increases for the obvious reason that the resilience of both states is comparable. In addition to resilience and dispersal, the *size* of the disturbed area is also important in determining whether a disturbance will recover (Figure A6 B). In general, a larger size perturbation increases the probability of expansion to a systemic shift. Again, if the dispersal rate is low, no recovery is more likely. An important overall result is that the probability of an induced global collapse is highest at intermediate levels of dispersal rate (Figure A6 A, B). At a low dispersal rate there are increased chances of no recovery. At a higher dispersal rate the chances of recovery increase. This is because the disturbed area effectively interacts stronger with the neighboring area and the disturbed area is rapidly ‘diluted’ over the rest of the landscape.


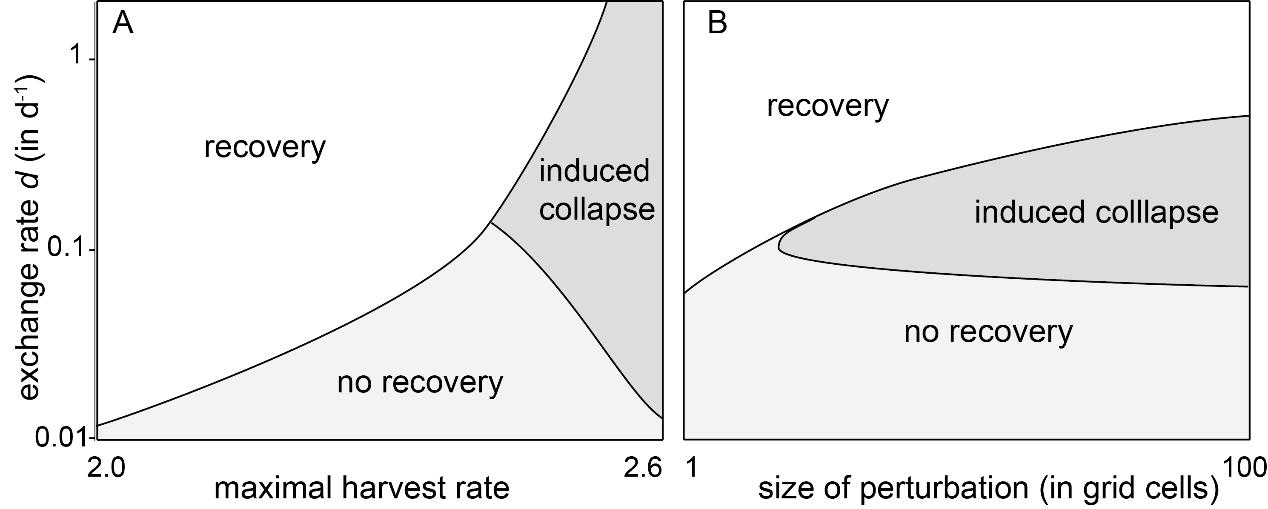


**Figure A6.** Recovery of local disturbance experiments in a lattice with local exchange between grid cells and their four neighboring cells. **(A)** Combined effects of resilience (in terms of the maximal harvest rate (*c*)) and local exchange rate *d* (with a fixed perturbation size: 7x7 cells) **(B)** Combined effects of perturbation size and local exchange rate *d* (with a fixed harvest rate *c*: 2.45 d^-1^). Note that the dispersal rate is on a log scale.
